# Evaluation of the Effect of a Single Intra-articular Injection of Allogeneic Neonatal Mesenchymal Stromal Cells Compared to Oral Non-Steroidal Anti-inflammatory Treatment on the Postoperative Musculoskeletal Status and Gait of Dogs over a 6-Month Period after Tibial Plateau Leveling Osteotomy: A Pilot Study

**DOI:** 10.3389/fvets.2017.00083

**Published:** 2017-06-08

**Authors:** Mathieu Taroni, Quentin Cabon, Marine Fèbre, Thibaut Cachon, Nathalie Saulnier, Claude Carozzo, Stéphane Maddens, Fabrice Labadie, Clément Robert, Eric Viguier

**Affiliations:** ^1^Small Animal Surgery Department, VetAgro Sup, Marcy L’Etoile, France; ^2^UPSP 2016A104, ICE, Interaction Cells Environment, Campus Veterinaire VetAgro Sup, Université de Lyon, Marcy l’Etoile, France; ^3^Vetbiobank SAS, Marcy L’Etoile, France

**Keywords:** mesenchymal stem cells, neonatal, dog, cranial cruciate ligament rupture, tibial plateau leveling osteotomy, allogeneic, knee surgery, lameness

## Abstract

**Objective:**

Compare the clinical and pressure walkway gait evolution of dogs after a tibial plateau leveling osteotomy (TPLO) for a cranial cruciate ligament rupture (CrCLR) and treatment with either a 1-month course of non-steroidal anti-inflammatory drugs (NSAIDs) or a single postoperative intra-articular (IA) injection of allogeneic neonatal mesenchymal stromal cells (MSCs).

**Study design:**

Prospective, double-blinded, randomized, controlled, monocentric clinical study.

**Animals:**

Sixteen client-owned dogs.

**Materials and methods:**

Dogs with unilateral CrCLR confirmed by arthroscopy were included. Allogeneic neonatal canine MSCs were obtained from fetal adnexa retrieved after C-section performed on healthy pregnant bitches. The dogs were randomly allocated to either the “MSCs group,” receiving an IA injection of MSCs after TPLO, followed by placebo for 1 month, or the “NSAIDs group,” receiving IA equivalent volume of MSCs vehicle after TPLO, followed by oral NSAID for 1 month. One of the three blinded evaluators assessed the dogs in each group before and after surgery (1, 3, and 6 months). Clinical score and gait and bone healing process were assessed. The data were statistically compared between the two groups for pre- and postoperative evaluations.

**Results:**

Fourteen dogs (nine in the MSCs group, five in the NSAIDs group) completed the present study. No significant difference was observed between the groups preoperatively. No local or systemic adverse effect was observed after MSCs injection at any time point considered. At 1 month after surgery, bone healing scores were significantly higher in the MSCs group. At 1, 3, and 6 months after surgery, no significant difference was observed between the two groups for clinical scores and gait evaluation.

**Conclusion:**

A single IA injection of allogeneic neonatal MSCs could be a safe and valuable postoperative alternative to NSAIDs for dogs requiring TPLO surgery, particularly for dogs intolerant to this class of drugs.

## Introduction

Cranial cruciate ligament rupture (CrCLR) in dogs is one of the most common orthopedic diseases in veterinary medicine (1), leading to stifle joint instability and inflammation and inevitably resulting in the development of stifle osteoarthritis (OA) (2–4). Treatment involves surgical joint stabilization and the medical management of pain and inflammation. To restore joint stability, tibial plateau leveling osteotomy (TPLO) is one of the most common surgical procedures performed worldwide (4–6), delivering quick and good clinical outcomes for a majority of the treated dogs (7–11). The biomechanical purpose of TPLO is to decrease the tibial slope angle through radial proximal tibial osteotomy, so the joint stability during gait no longer relies on the integrity of the cranial cruciate ligament.

Immediate postoperative management includes non-steroidal anti-inflammatory drugs (NSAIDs) to alleviate pain and inflammation resulting from both preoperative CrCLR-related arthritis and the surgical procedure (11, 12).

However, some patients cannot be treated with NSAIDs either because they developed intolerance with gastrointestinal symptoms or because of concurrent gastrointestinal bleeding, impaired renal, hepatic, cardiac function, or hemorrhagic disorders (13). Alternative but less effective treatments, such as tramadol as a sole medication, are commonly used to replace NSAIDs in postoperative management with variable outcomes (14).

In recent years, various clinical studies have reported mesenchymal stromal cells (MSCs) as a promising therapeutic approach for managing inflammation and pain in patients with joint disorders (15, 16), patients with spinal cord injury (17), and patients suffering from rheumatoid arthritis (18). Even if the specific molecular mechanisms by which MSCs alleviate pain remain undefined, numerous evidence suggest that the analgesic effects of MSCs could reflect anti-inflammatory activity. MSCs generate a local immunosuppressive microenvironment by secreting cytokines (19). In addition to immunomodulatory functions, MSCs secrete trophic and soluble factors that reduce synovial inflammation, exert anti-catabolic effects, and recruit endogenous MSCs in the joint environment (20–23). Taken together, these pleiotropic actions justify an evaluation of the extent to which the local administration of MSCs compares to NSAIDs in managing postoperative joint pain in dogs (24).

The context of a perioperative administration during TPLO implies the rapid availability of MSCs between diagnosis and surgery, arguing for the use of ready-to-use allogeneic cell products. This approach, mainly based on adipose tissue-derived MSCs, has been demonstrated as clinically safe in several clinical studies in humans (25), dogs (26), and horses (27). A few studies aimed at comparing the safety and efficacy of allogeneic vs autologous MSC in horses, showing controversial results, depending on MSC’s tissue origin. Carrade and colleagues reported no difference between treatment groups, in regard to vital parameters, joint swelling, or lameness, suggesting that allogeneic administration of MSC into normal joint does not elicit a greater inflammatory response than autologous injection (28). Other studies, performed with allogeneic or autologous bone marrow-derived MSCs IA transplantation, reported a greater acute inflammatory joint response with the use of non-self cells (29), which could result in adverse clinical reaction after a second injection (30). This may suggest that MSCs do not elicit the same degree of joint inflammation, depending on their tissue origin. One of the main concerns following allogeneic MSC transplantation is the production of allo-antigens, which could induce immune response if a second injection is performed. Controversy exists in the literature, as some groups reported that allogeneic MSC may not induce a detectable immune response after IA injection (28, 31), while others showed that allogeneic MSCs are capable of eliciting antibody responses *in vivo* (30, 32). It is important to note that these studies, except Carrade’s work, were realized with bone marrow MSCs from adult donors. It could not be excluded that MSCs induce various degree of immune response in the recipient depending on whether they are retrieved from adult or neonatal tissues, as suggested by *in vitro* data showing that neonatal MSCs generate lower immune response than their adult counterparts (33). This warrants further work, as it is now established that MSCs cannot be considered as truly immune privileged, but more immune evasive, and that environmental factors, such as inflammation affect their immunogenicity (34).

To our knowledge, no study has evaluated the clinical effect of perioperative intra-articular (IA) injection of allogeneic neonatal MSCs in dogs undergoing TPLO. The objective of the present study was to evaluate an alternative postoperative management protocol to the standard NSAIDs, based on a single IA injection of allogeneic neonatal MSCs immediately postoperative. Our null hypothesis was that dogs receiving one intraoperative IA injection of umbilical-derived MSCs after TPLO to treat CrCLR, without postoperative NSAIDs treatment, exhibited no difference on the postoperative outcome (up to 6 months) compared to dogs receiving IA MSC-vehicle injection and NSAID treatment for 1 month.

## Materials and Methods

### Study Design

The present study was a prospective, randomized, controlled, double-blinded study conducted at the small animal hospital of the veterinary campus of VetAgro Sup. The protocol was approved by the ethical committee of VetAgro Sup (no. 1415).

### Inclusion and Exclusion Criteria

The animals included in the present study were client-owned dogs presented for a natural occurring unilateral CrCLR and weighing between 20 and 60 kg. CrCLR was diagnosed at the clinical examination. NSAIDs or corticosteroids were stopped at least 1 week prior to initial evaluation, or 6 months prior to initial evaluation if the dogs had been injected with sustained-release corticosteroids. The owners signed an informed consent form prior to the enrollment of their animals in the present study. The exclusion criteria were the presence of a concomitant orthopedic condition or a concomitant systemic disease. Study dropout criteria were local infection at the site of surgery, major complication requiring surgery, NSAIDs or corticosteroids requirement, unexpected inflammatory reaction after surgery or contralateral CrCLR occurring during the study period.

### Treatment Protocol

Eligible animals were randomly assigned to two groups through a clinical pilot study conducted independently from clinicians. The dogs in “MSCs group” received in a blinded fashion 10 × 10^6^ MSCs in the operated stifle joint by a single injection in the arthroscopic portal site before closure of the joint, according to Nganvongpanit et al. (35) and Fajardo et al. (36). Dogs receiving MSCs were prescribed them from the day after surgery and for 1 month thereafter, with an “A” treatment corresponding to an AINS placebo with no claim for any anti-inflammatory or an OA management in the product datasheet. The dogs in the “NSAIDs group” received the MSC vehicle (phosphate-buffered saline; PAN Biotech, Germany) at the same time of surgery as dogs in the “MSCs group” and were prescribed with a “B” treatment corresponding to NSAIDs (firocoxib, 5 mg/kg, orally, once daily) for 1 month, starting the day after surgery. Both A and B treatments have a similar pharmaceutical presentation and were delivered at discharge by an in-house specialized pharmacist, independently from surgeons.

### Pre- and Postoperative Care

The initial diagnosis of CrCLR was made clinically and subsequently confirmed arthroscopically. Stifle arthroscopy also enabled meniscal evaluation, and partial meniscectomy was performed in case of concurrent meniscal tear. Contralateral stifle evaluation consisted in orthopedic examination and orthogonal radiographs to rule out bilateral CrCLR. Affected stifle was subsequently stabilized using a TPLO procedure (TPLO plate, Synthes, Switzerland) according to Kowaleski et al. (9). Board-certified surgeons with experience in both stifle arthroscopy and surgery (Eric Viguier, Thibaut Cachon, and Claude Carozzo) performed the stifle arthroscopy and TPLO. Anesthetic and analgesic protocols were standardized: the dogs were premedicated with the administration of morphine [0.4 mg/kg, intramuscularly (IM)] and acepromazine (0.03 mg/kg, IM). After the introduction of a cephalic intravenous catheter, anesthesia was induced with ketamine [5.0 mg/kg, intravenously (IV)] and diazepam (0.2 mg/kg, IV) and maintained with isoflurane in 100% O_2_ after orotracheal intubation. A preoperative femoro-sciatic nerve block was performed with ropivacaïne (2.0 mg/kg) using electrostimulation. During surgery, repetitive IV fentanyl boli (1.0 µg/kg) was injected in the case of intraoperative pain (assessed as an increase in respiratory and heart rates), and IV morphine (0.2 mg/kg) was administered prior to extubation. Postoperative analgesia was controlled using IV morphine boli (0.1 mg/kg) depending on the postoperative pain score assessed using the 4A-Vet pain score (37) (Table 1). The dogs were maintained at the hospital for clinical evaluation for 3 days after surgery. All dogs received tramadol (3–5 mg/kg, orally, twice daily) for 10 days after discharge. If postoperative pain could not be managed with tramadol, the dog was excluded from the study, and a clinical evaluation was performed by the clinicians to provide an appropriate management (medical or surgical).

**Table 1:** French Veterinary Association for Anaesthesiology and Analgesia (4A-Vet) multiparametric scoring scale.

Subjective overall assessment	Absence of pain	0
		1
		2
	Intolerable pain	3

General behavior	Among the following symptoms:	
	Shows respiratory alterations	◻
	Vocalizing	◻
	Crouched/stooped posture	◻
	Unable to move	◻
	Restless and/or depressed	◻
	Loss of appetite	◻
	Looks at, chews/licks the surgical site	◻
	Lame, moves about with difficulty or is reluctant to move about	◻
	– No sign present – Only 1 sign present – 2–4 signs present – 5–8 signs present	0 1 2 3

Interactive behavior	Is attentive and responds to touch/voice	0
	Timid/nervous response	1
	Does not respond immediately	2
	Does not respond or responds aggressively	3

Heart rate	<10% increase	0
	11–30% increase	1

Initial value	31–50% increase	2
	>50% increase or cannot be assessed	3

Reaction at palpation or manipulation of the surgical site	No visible or audible response	
	– after 4 tests	0
	Visible or audible response(s)	
	– at the fourth – at the second and third – at the first test	1 2 3

Intensity of the response	No response	0
	Responds easily, tries to escape	1
	Turns head or vocalizes	2
	Aggressive response or non-responsive	3

Total score	1–5: slight pain	
	6–10: moderate pain	
	11–18: severe pain	

### MSC Preparation

Canine MSCs were kindly provided by Vetbiobank (Marcy l’Etoile, France). Briefly, MSCs were isolated from neonatal tissue collected during C-sections from five bitches. The screening of prevalent microbiological pathogens was performed from a blood sample collected from each bitch to qualify their microbiological status. Briefly, collected tissue was extensively washed with 0.9% NaCl, then submitted to mechanical and enzymatic digestion in two steps, as previously described (38). Cells were amplified in a proprietary medium in tissue culture flasks and harvested after passages 3–4. Cells were cryopreserved and stored in a liquid nitrogen Dewar until use. Characterization of each cell product was realized as previously described (38) (Figure S1 in Supplementary Material). Briefly, cells display conventional phenotype of canine MSCs (CD44+; CD29+; CD90+; MHC2−; CD45−; CD34−), differentiate into the three mesodermal lineages *in vitro* (adipogenic, osteogenic, and chondrogenic), and express indoleamine 2,3-dioxygenase upon exposure to interferon-gamma stimulation (Additional file). Sterility testing was also performed on every cellular product.

The day of the surgery, MSCs were thawed, washed with Dulbecco’s phosphate-buffered saline (D-PBS; Pan Biotech) three times, and cell viability was assessed by Trypan blue exclusion assay (average viability upon thawing was 89%). At least 10 × 10^6^ viable MSCs were homogenized in 300 µL of D-PBS and aseptically conditioned in 1 mL syringe with a 20 gauge needle. A technician, independent from the medical team, homogenized the cell product prior to injection to ensure that the surgeon remained unaware of the nature of the treatment. The MSCs were injected within 6 h after thawing.

### Clinical Evaluations

Preoperative radiographic scoring was performed on each affected stifle joint to assess the OA extent in eligible dogs. The preoperative evaluation of dogs the day of surgery (D0) and during follow-up visits at 1 month ± 4 days (M1), 3 months ± 4 days (M3), and 6 months ± 4 days (M6) composed a clinical score and gait evaluation by a single blinded evaluator. The clinical score was postoperatively assessed at D1, D2, and D3. Follow-up examinations at M1, M3, and M6 included the radiographic evaluation of bone healing (see below). The standard postoperative follow-up procedure in the hospital includes an informal telephone call to the owner at 1–2 weeks after surgery to fetch general information about the recovery of the dog. Even though it is not a strict and thorough evaluation, information was available for all the dogs and is given for information purpose only.

#### Radiographic Scoring

Two board-certified surgeons blindly performed preoperative OA assessment on preoperative stifle joint radiographs (D0) according to a published score (39). The readings were repeated three times for each radiograph, and the mean score was calculated.

Two of the board-certified surgeons blindly performed the bone healing assessment on follow-up radiographs (M1, M3, and M6) according to a published score (40). This score utilizes a 10-point scale, with 10 assigned to a completely healed and remodeled bone. The readings were repeated three times for each radiograph, and the mean score was calculated.

#### Orthopedic Pain Scoring

One of the three experienced board-certified surgeons blindly performed the clinical score determination at D0, D1, D2, D3, M1, M3, and M6. The evaluated variables included lameness at walk, pain upon stifle palpation, pain upon stifle manipulation, and local heat (Table 2). Each of four variables was graded from 1 to 4, leading to a clinical score from 4 to 16 (the highest score reflecting the worst clinical condition).

**Table 2:** Orthopedic pain scoring system.

Parameter	Notation	
Lameness	1	No lameness
	2	Intermittent weight-bearing lameness
	3	Permanent weight-bearing lameness
	4	Non-weight-bearing lameness

Pain	1	No pain
Palpation-pression	2	Mild
	3	Moderate
	4	Severe

Pain	1	No pain
Mobilization	2	Mild
	3	Moderate
	4	Severe

Heat	1	No warmer than contralateral limb
	2	Mildly warmer
	3	Moderately warmer
	4	Markedly warmer

Total score	Lameness + pain (palpation-pression + mobilization) + heat	

#### Semiquantitative Gait Analysis

A blinded operator performed the gait evaluation using a pressure walkway (GaitRite^®^, Biometrics, France) (41, 42). Each dog was acclimated to the room and the walkway for 5 min. The run was considered valid if the dog walked on the pressure walkway at a constant walk speed, at a walking pace, with a relaxed and regular gait and without pulling on the leash. Three valid runs of six gaits were required for each dog. The data collected to evaluate the gait included the ratio between the front and hind limbs for total pressure (FH/TP), ratio between the front and hind limbs for number of sensors activated (FH/NS), Symmetry index between non-affected and operated limbs for stance percentage during gait cycle (SI/%), symmetry index between non-affected and operated limbs for total pressure (SI/TP) and symmetry index between non-affected and operated limbs for number of sensors (SI/NS). The mean values and SDs for the three valid passes were calculated.

### Statistical Analysis

Statistical analysis was performed using GraphPad Prism 6.0 (GraphPad Software, USA). Baseline comparisons between MSCs and NSAIDs groups on clinical score, weight, age, meniscal tear, and radiographic score were examined using the Mann–Whitney *U* test. Categorical data (gender) were compared using Fisher’s exact test. Changes in the score over time for each parameter in each group were examined using the non-parametric Friedman repeated measures ANOVA on rank sum test. *Post hoc* comparisons were made using Dunn’s test.

A comparison of the responses (clinical score, bone healing score, and gait evaluation) between the MSCs and NSAIDs groups was conducted using two-way repeated measures ANOVA, with treatment and time as grouping variables. A *post hoc* comparison was obtained using Sidak’s test.

A *P*-value < 0.05 was accepted as statistically significant for all tests.

## Results

### Clinical Information for the Study Cohort

Twenty client-owned dogs met the inclusion criteria and were enrolled in the present study. Fourteen dogs completed the present study (nine in the MSCs group and five in the NSAIDs group). One dog was dropped from the study after enrollment for reasons unrelated to the tested product (NSAIDs group); two dogs were dropped from the study because of postoperative infection at the site of surgical implant, both from the NSAIDs group; and three dogs were dropped from the study because of tendonitis or pain in the contralateral limb requiring NSAID treatment (two dogs from the CSM group) or surgical treatment (one dog from the NSAIDs group).

For the dogs completing the study, the ages ranged from 1 to 13 (median value = 5), body weight values ranged from 21 to 53.2 (median value = 32.4), body condition values ranged from 1.5 to 3.5 (on a 5-points scale, median value = 3), with eight females and six males (Table 3). There was no significant difference at D0 between the groups in age (*P* = 0.08), body weight (*P* = 0.71), body condition (*P* = 0.09), gender distribution (*P* = 0.51), or meniscal tears diagnosis (*P* = 0.064).

**Table 3:** Inclusion data of the dogs.

No.	Group	Affected joint	Breed	Sex	Weight	Age	Body condition
1	Non-steroidal anti-inflammatory drug (NSAID)	Right	Crossbreed dog	F	27.5	8	3
2	NSAID	Right	Crossbreed dog	F	25.1	10	3.5
3	Mesenchymal stromal cell (MSC)	Left	Leonberger	F	52	4	1.5
4	MSC	Left	Boxer	M	32	5	3
5	MSC	Right	Cane corso	F	41	2	3
6	MSC	Right	Bracco	M	24.9	8	3
7	MSC	Right	Labrador retriever	F	25.7	3	3
8	NSAID	Left	Bernese mountain	M	53.2	7	3.5
9	NSAID	Right	Labrador retriever	F	32.3	5	4
10	MSC	Left	Cane corso	M	58	3	3
11	MSC	Left	Labrador retriever	M	41.3	3	4
12	NSAID	Left	Labrador retriever	F	32.5	7	4.5
13	MSC	Right	German shepherd	F	21	13	3
14	MSC	Left	Cane corso	M	42	7	3

*Weight values are in kilograms and age values in years*.

Osteoarthritis radiographic scores at D0 were also not significantly different among MSC and NSAIDs groups (*P* = 0.46). There were no significant differences in terms of clinical score and between groups (*P* = 0.71). The median clinical scores (maximum score of 16) were nine for MSCs and NSAIDs groups. At baseline (D0), no difference between MSCs and NSAIDs groups was observed on either FH/TP (*P* = 0.52), FH/NS (*P* = 0.35), SI/% (*P* = 0.6), SI/TP (*P* = 0.61) or SI/NS (*P* = 0.61). All baseline clinical parameters are detailed (Table 4).

**Table 4:** Preoperative characteristics of the dogs enrolled in the present study.

		Control (*N* = 5)	Mesenchymal stromal cell (MSC) (*N* = 9)	*P*-value
Epidemiological data	Weight	34.12 ± 11.13	37.54 ± 12.64	0.71
	Age	7.4 ± 1.82	5.33 ± 3.5	0.08
	Gender	4 females/1 male	4 females/5 males	0.51
	Meniscal tear	5 yes/0 no	3 yes/6 no	0.06

Clinical score		9.4 ± 1.82	8.67 ± 1.22	0.53

Osteoarthritis radiographic score		11.36 ± 4.98	9.37 ± 3.67	0.46

Gait evaluation	FH/TP	1.91 ± 0.23	1.82 ± 0.27	0.89
	FH/NS	1.23 ± 0.17	1.5 ± 0.14	0.48
	SI/%	0.76 ± 0.10	0.87 ± 0.06	/
	SI/TP	0.44 ± 0.20	0.58 ± 0.17	0.34
	SI/NS	0.56 ± 0.19	0.67 ± 0.13	0.25

*All values represent the means ± SD, except for gender distribution and meniscal tears. No significant difference was observed among the non-steroidal anti-inflammatory drugs and MSCs groups for any parameter*.

### Clinical Evaluation

**Figure 1:**
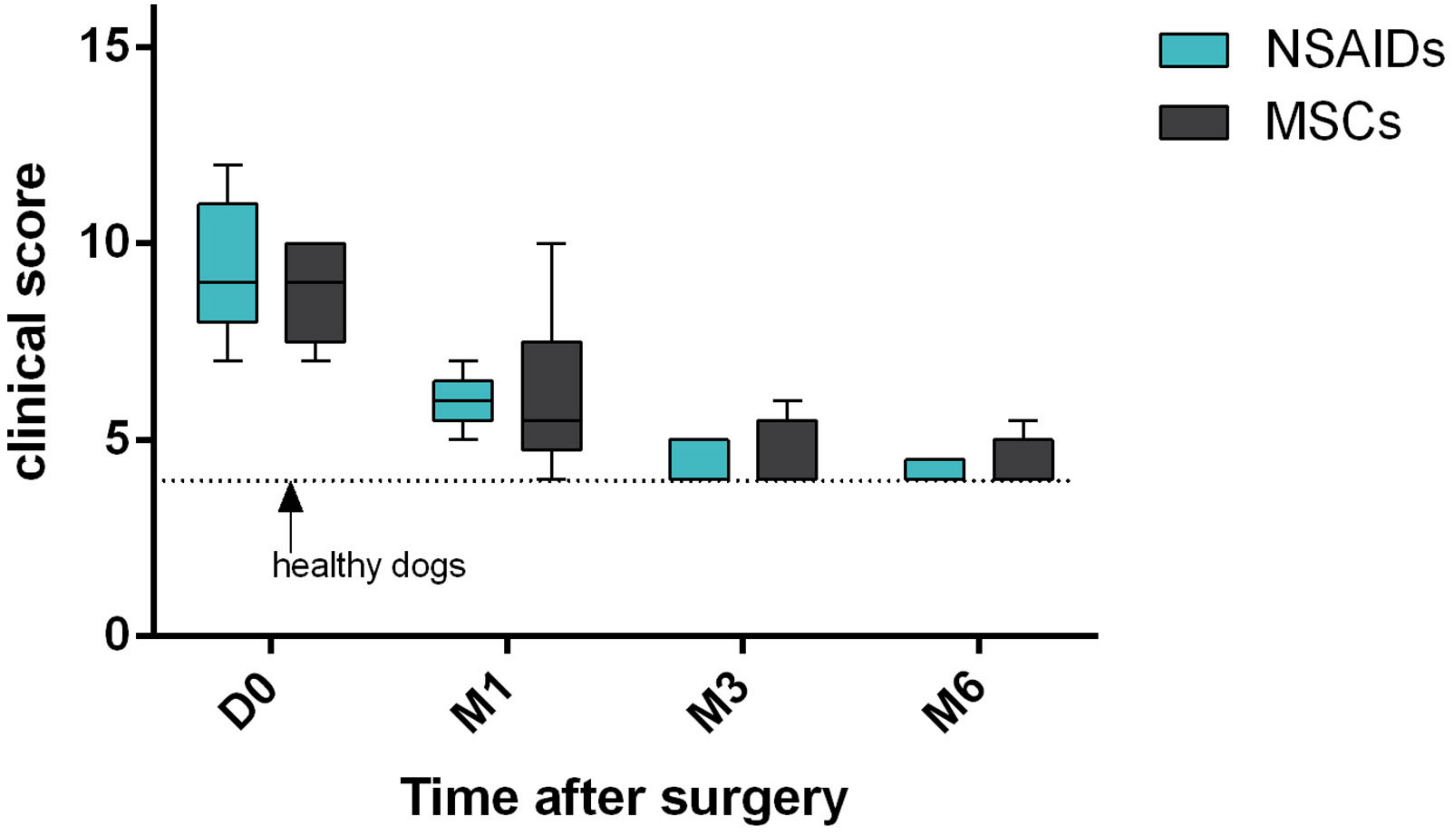
Clinical scores’ comparison between “mesenchymal stromal cells (MSCs)” group and “non-steroidal anti-inflammatory drugs (NSAIDs)” group. No significant difference was observed. Larger distribution was observed in the “MSCs” group.

The clinical score significantly improved compared to D0 in both groups at M3 (*P* = 0.04 for NSAIDs group and *P* = 0.02 for MSCs group) and M6 (*P* = 0.007 for NSAIDs group and *P* = 0.01 for MSCs group) (Figure 1). The clinical score did not show a significant difference between the two groups at D1 (*P* = 0.97), D2 (*P* = 0.61), and D3 (*P* = 0.70) and at M1 (*P* = 0.99), M3 (*P* = 0.99), and M6 evaluations (*P* = 0.98) (Table 5).

**Table 5:** Postoperative comparison of the clinical and bone healing scores between both groups.

		Control	Mesenchymal stromal cell (MSC)	*P*-value
Clinical score	M1	6 ± 0.71	6.1 ± 1.92	0.99
	M3	4.6 ± 0.55	4.6 ± 0.86	0.99
	M6	4.2 ± 0.27	4.5 ± 0.61	0.98

Bone healing radiographic score	M1	3.6 ± 1.78	6.1 ± 2.2	0.008
	M3	8.2 ± 1.39	9.5 ± 1.15	0.28
	M6	9.8 ± 1.27	10 ± 0	0.99

*All values represent the means ± SD. No significant difference was observed for clinical scores among the non-steroidal anti-inflammatory drugs and MSCs groups at any evaluation time. A significant difference was observed at M1 between groups for bone healing*.

During informal telephone interview of the owner made 1–2 weeks after surgery, seven owners (four in the MSCs group and three in the NSAIDs group) reported mild transient lameness occurring after discharge that did not require further medication. Apart from these, no local or systemic adverse effect was observed after MSC injection during the course of the study, and no side effects were observed in dogs receiving NSAIDs medication.

### Bone Healing

**Figure 2:**
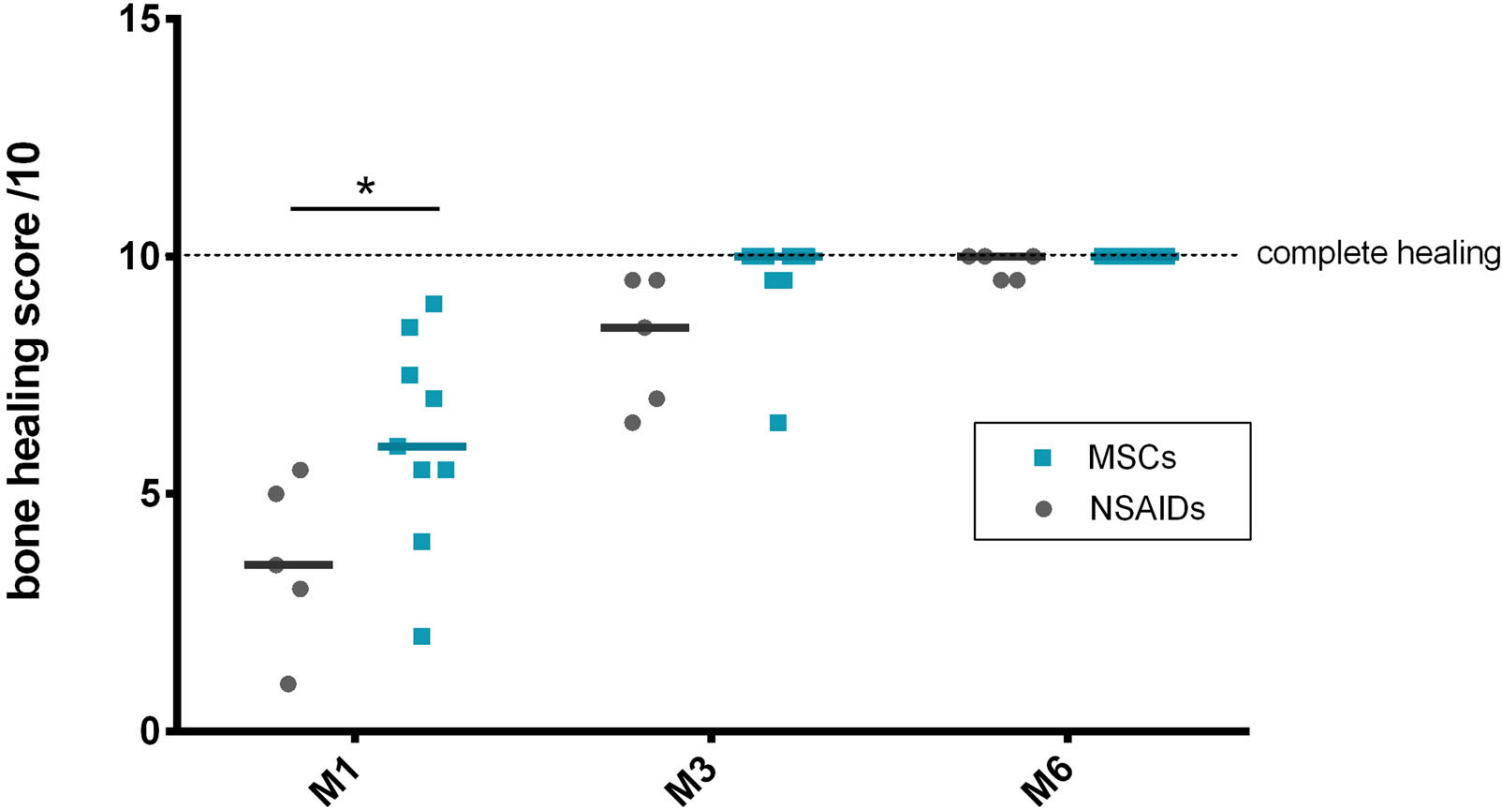
Bone healing scores’ comparison between the “mesenchymal stromal cells (MSCs)” and “non-steroidal anti-inflammatory drugs (NSAIDs)” groups. A significant difference was observed at M1 between the groups.

The radiographic evaluation score for bone healing was significantly higher in the MSCs group at M1 (*P* = 0.0001). No significant difference was detected at M3 (*P* = 0.0662) or M6 (*P* = 0.99) between the groups (Figure 2). At M3, six dogs had complete healing (10/10) in the MSCs groups with none in the NSAIDs group.

### Gait Evaluation

**Figure 3:**
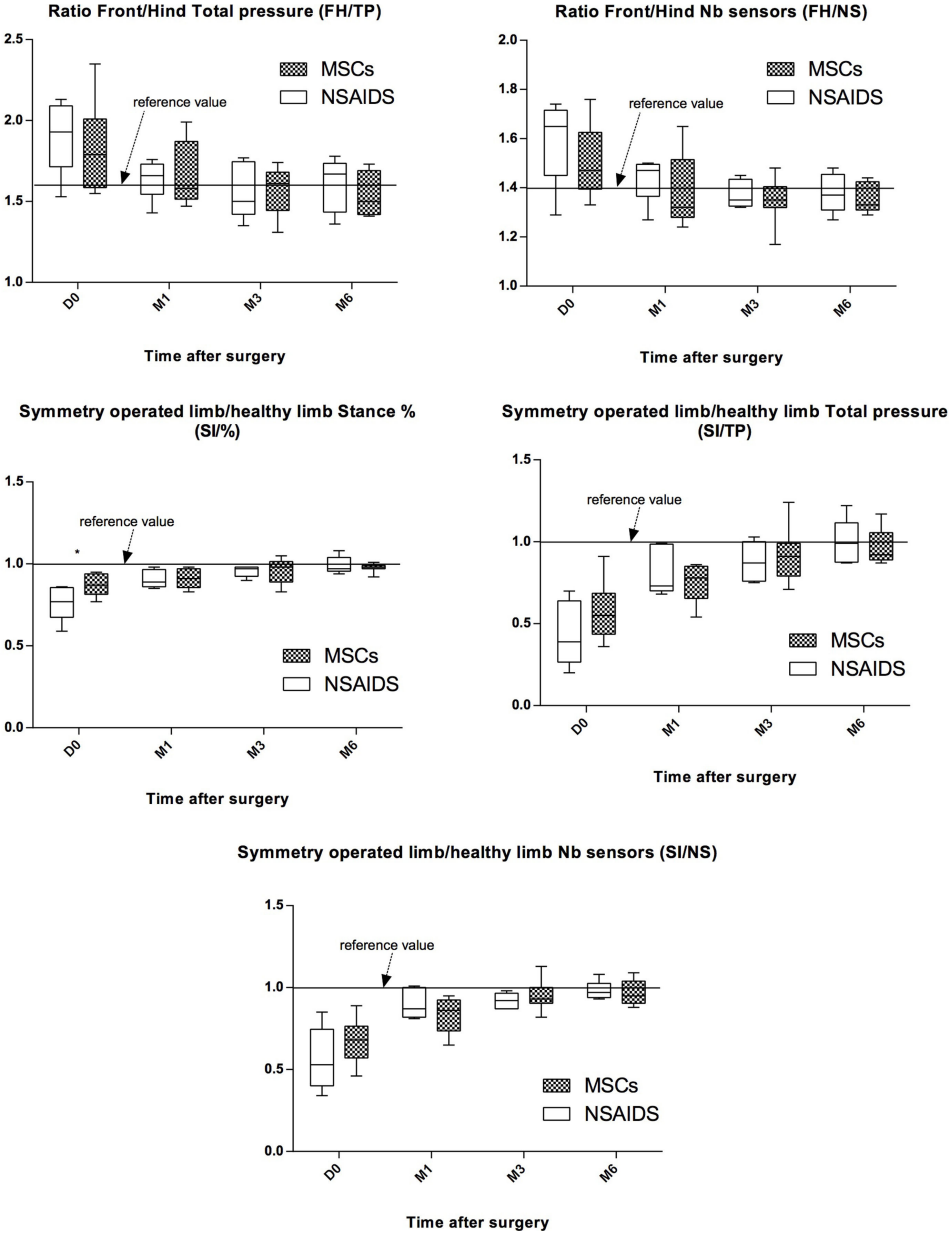
Evolution of pressure walkway values between preoperative (D0) and postoperative (M1, M3, and M6) evaluations for both groups for the five studied parameters. Black line represents reference values observed in healthy animals. All values represent the means ± SD. Improvement was observed in both groups for the five studied parameters. No significant difference was observed between the non-steroidal anti-inflammatory drugs (NSAIDs) and mesenchymal stromal cells (MSCs) groups for any parameter after D0.

Gait evaluation was performed for all dogs at D0 and at the M1, M3, and M6 follow-up visits. At D0, no significant difference between the MSCs and NSAIDs groups was observed, except for SI/% (*P* = 0.01). At M1, M3, and M6, no significant difference among the MSCs and NSAIDs groups was detected for the different ratios (Figure 3). The weight distribution between forelimbs and hindlimbs (FH/TP and FH/NS ratios) progressively normalized to the reference values over time (acceptable values at M3 for both groups). Symmetry indices (SI), representing weight distribution changes between the operated and the contralateral control limb showed the same evolution with improvement and return to normal values over time (acceptable values at M3 for both groups), without significant differences between the two groups at M1, M3, and M6. Using the ratios (M1–D0)/reference, (M3–D0)/reference, or (M6–D0)/reference, no significant difference was observed for lameness improvement between the two groups regarding gait evaluation for the five studied parameters at M1, M3, and M6 evaluation.

### Statistical Power

Statistical power was calculated to verify whether the non-significant results reflected a lack of a relationship between the groups or a lack of statistical power. We calculated 87% to detect a difference of 0.2 on the analysis of the GaitRite^®^. We calculated 70% to detect an equal to or superior difference of 2 on the clinical score.

## Discussion

Non-steroidal anti-inflammatory drugs represent a pivotal class of therapeutic agents against inflammation and pain and are routinely administered following invasive surgery to improve animal well-being (43). Nonetheless, the long-term use of NSAIDs is not recommended for a variety of patients, warranting the development of valid therapeutic alternatives, such as MSCs recently used for their anti-inflammatory and antinociceptive activity in an experimental inflammatory model (44). In the present study, neonatal MSCs were evaluated as an alternative to NSAIDs in the management of the pain and walk recovery of dogs operated using TPLO for CrCL rupture.

### Clinical Significance

Overall, this clinical trial did not reveal any difference in clinical scores, gait evaluation, and lameness improvement between dogs receiving either one single IA articular injection of canine allogeneic neonatal MSCs at the time of the surgery or a 1-month course of oral NSAIDs after a TPLO procedure. Clinical evaluation using a scoring system revealed adequate pain management for dogs treated using both approaches. No significant difference was observed for any parameters at the M1, M3, and M6 follow-up time points. An objective, semiquantitative assessment of lameness was performed in the present study using a pressure walkway system (GaitRite^®^). This technique has been validated to measure the spatiotemporal gait characteristics of healthy animals and is commonly used to assess gait modifications resulting from orthopedic or neurologic conditions in dogs (41, 42, 45–47). The results from the walkway system showed no difference in either the forelimb or hindlimb total pressure ratio and the symmetry index between MSCs and NSAIDs groups at any time of the trial. This finding enables the objective comparison of both approaches without suffering from the bias introduced by the evaluator when using standard gait evaluation, suggesting that MSCs do not induce gait worsening.

### Clinical Model/Study Design

This study aims to compare two different approaches of the postoperative period. Both approaches rely on different mechanisms of action of the therapeutic agents, different modes of administration and pharmacokinetics, as it is well recognized that a short to medium course of NSAID may be sufficient to get satisfactory effect, which extends beyond the total washout of the product (11). Clinical and functional follow-up were therefore realized during several months after NSAID discontinuation to evaluate short and long-term effects. CrCLR surgical treatment was selected to examine the study hypothesis, as CrCLR is one of the most frequent orthopedic conditions in dogs (1). CrCLR management through surgery has been well accepted, even if the type of stabilization remains a matter of debate (48). Clinical studies performed with client-owned animals are powerful, as these studies integrate all “real life” variables that may not be considered in studies performed with an experimental model, enabling the translation of the results to the field with a good level of confidence (49). The present study was designed to minimize all sources of variation (see Limitations). After randomization, the groups were similar in gender, age, body weight, body condition, clinical and preoperative radiographic scores, and presence of meniscal tears, and therefore could subsequently be compared. Trained surgeons with extensive experience with this procedure performed the stifle arthroscopy and TPLO according to Kowaleski et al. to decrease variability between dogs in terms of pain and joint inflammation (9). Stifle arthroscopy was preferred over stifle arthrotomy to better assess meniscal tears and decrease joint inflammation and pain in the postoperative period (50, 51). Furthermore, anesthetic and intraoperative and postoperative analgesic protocols were standardized. All precautions were taken to obtain as much homogeneity as possible and improve comparison between groups.

### Osteotomy Healing

**Figure 4:**
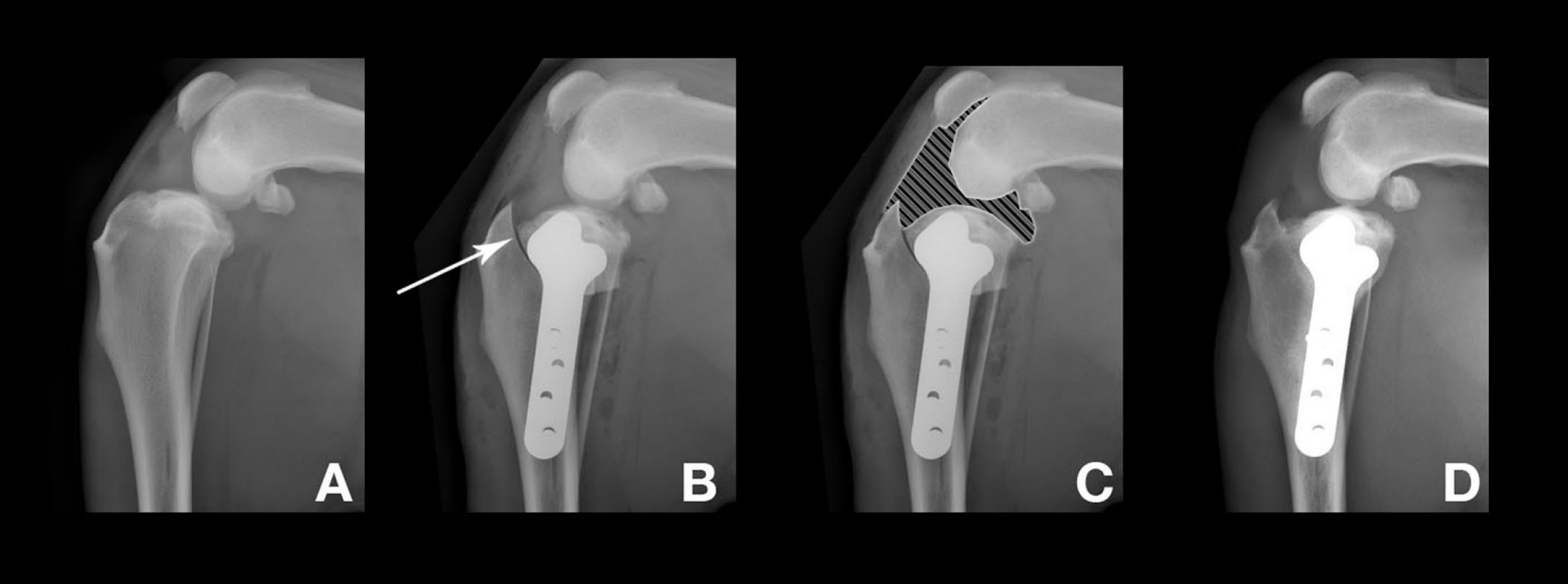
Radiographs before and after the tibial plateau leveling osteotomy surgery. The dog presented here was in the “mesenchymal stromal cell” group. **(A)** Preoperative stifle joint. **(B)** 1 day after surgery. Arrow: osteotomy line. **(C)** 1 day after surgery. Hatched area: articular zone. Note the direct contact with the osteotomy line. **(D)** M3 after surgery, complete bone healing and remodeling.

The TPLO procedure, as briefly presented above, implies an osteotomy, rotation, and stabilization of the proximal tibia to transform the stifle joint biomechanics. Bone healing is one of the main problems with TPLO, and although conflicting results have been published (11, 52), the use of NSAIDs may delay the bone healing process. In the present study, no delay in bone healing was observed in either of the two groups, and the osteotomy line disappeared within a typical 1-month period. However, a significant difference in bone healing radiographic scores was observed at M1 evaluations. As a result of the TPLO technique, MSCs and their secreted substances contained in the synovial fluid come in contact with the osteotomy site (Figure 4) and could improve bone healing (53).

Based on these results, we could not conclude whether NSAID medication delays bone healing or whether MSCs improve bone healing time; however, the results clearly show a benefit of MSCs over NSAID on this particular point. Further investigation and a dedicated study are needed to address this specific point. Indeed, an improvement of the bone healing time is clinically relevant and may reduce the convalescence period and allow an early return to activity.

### MSC Sources

Mesenchymal stromal cells are mainly derived from three sources: bone marrow, adipose tissue, or fetal adnexa. The present study is the first to describe the use of canine allogeneic neonatal MSCs in dogs. This source of MSCs is of great interest since the cells are recovered at the end of C-section and do not require any surgical sampling, in contrast to adipose tissue and bone marrow-derived MSCs (54–56). Neonatal MSCs differ from adult MSCs with regard to their biological properties, particularly as neonatal MSCs are spared from proaging factors that significantly decrease MSCs regenerative potential (57–59). A body of *in vitro* evidences suggests also that neonatal MSCs are less immunogenic than adult MSCs and produce high levels of immunomodulatory molecules, even if *in vivo* data are not yet available to extrapolate these results (33, 60). One of the other interesting features of neonatal MSCs is the potential generation of banks of allogeneic MSCs from fetal adnexa donors, without requiring any medical or surgical gesture on healthy animal donors to recover the cells. A cryogenic storage step ensures the availability of cell product and facilitates more extensive laboratory quality control tests to ensure microbiological safety and cell characteristics. The standardization of cell products also increases the probability of obtaining a reproducible clinical result. These observations make fetal adnexa a promising source of MSCs for clinical use, including joint inflammation and OA management in dogs.

The clinical safety of autologous and allogeneic IA MSCs injection has been previously reported in dogs with Ad-MSCs after hip or elbow injections (54, 61) and was recently confirmed in a clinical study using client-owned dogs affected with OA (26). In the present study, no local acute side effect was observed after IA injection of canine allogeneic neonatal MSCs. In addition, no systemic effect was observed after IA injection of canine allogeneic neonatal MSCs for the entire study period of 6 months. These data reinforce the clinical safety of the use of allogeneic neonatal MSCs although this study was not designed to evaluate biological safety with the monitoring of donor immune response (29, 31, 61).

### Limitations

The present study relies on a single investigational site. Although a single investigational site confers standardization and reduces inter-sites variability regarding clinical practices, multi-centric clinical studies require the assessment of any bias related to a local practice, which may have influenced clinical outcome.

The number of dogs in this study was low and even if there was no statistical difference between the population of the two groups, there is a trend toward younger dogs with better body condition having better preoperative radiographic score.

Another limitation is the delay of the first clinical evaluation after TPLO. Indeed, despite an overall undeniable improvement, some of the patients experienced transient lameness a few days after discharge. Although this effect was observed in both groups, telephone conversations with the owners between discharge and M1 follow-up examination highlighted a trend of transient lameness more pronounced in the MSCs group. One explanation could be the delay in MSCs required to recover the full immunomodulatory effect (62, 63) after thawing with an optimum of 48 h. Additionally, the primary action of these cells is through paracrine mechanisms, contrary to NSAIDs, which act within 1–2 h after administration. Even if MSC therapy could be considered as an alternative therapy, improving the ease of use, treatment observance, or pharmaceutical side effects, NSAIDs could be helpful in the first few days after surgery to improve the well-being of the dog. In the end, rather than considering single medication strategies, a multimodal approach may be key to improving care in veterinary orthopedics.

### Perspectives

Despite joint stabilization, OA remains a concern in dogs with CrCLR (64). This condition could reflect either a preexisting OA condition or surgically induced cartilage trauma or both. The beneficial effects of IA injections of MSCs for OA management have been described for humans (16) and dogs (26), and it is reasonable to imagine a beneficial impact of MSCs on OA progression in dogs that underwent TPLO or any other stifle joint stabilization technique. Only a dedicated clinical study design may address this question.

## Conclusion

Single postoperative IA injection of canine allogeneic neonatal MSCs leads to level of postoperative lameness and pain outcome after TPLO not significantly different to ones obtained with NSAIDs systemic administration in dogs. These promising results are a first step to considering MSCs as an efficient alternative to long-term NSAID in the management of the postoperative pain after TPLO and may justify the use of these cells in veterinary medicine to widen the therapeutic arsenal of the clinician in the management of postoperative pain, ease treatment observance and avoid NSAIDs side effects.

## Ethics Statement

The protocol was approved by the ethical committee of VetAgro Sup (no. 1415).

## Author Contributions

The authors on this paper qualify as have providing the following overall contributions: (1) substantial contributions to the conception or design of the work; or the acquisition, analysis, or interpretation of data for the work, (2) drafting the work or revising it critically for important intellectual content, (3) final approval of the version to be published, and (4) agreement to be accountable for all aspects of the work in ensuring that questions related to the accuracy or integrity of any part of the work are appropriately investigated and resolved. Specifically, each authors’ contributions were as follows: SM, MF, NS, CR, and FL—conception and design, administrative support, and provision of study material; data collection, assembly, and analysis; and manuscript drafting. EV, TC, CC, QC, and MT—all acted as study site investigators and provided study design review, patients, evaluation of individual patients and adverse events, analysis, and interpretation, editing/review of manuscript drafts, and final approval of manuscript.

## Conflict of Interest Statement

SM is a current shareholder of Vetbiobank. MF, NS, CR, and FL were employed by Vetbiobank at the time of the study. There is no commercial or financial connection between Vetbiobank and the four co-authors (EV, TC, QC, and MT). CC declares no conflict of interest.
