# Supplementary figures and images for: Evaluation of the Effect of a Single Intra-articular Injection of Allogeneic Neonatal Mesenchymal Stromal Cells Compared to Oral Non-Steroidal Anti-inflammatory Treatment on the Postoperative Musculoskeletal Status and Gait of Dogs over a 6-Month Period after Tibial Plateau Leveling Osteotomy: A Pilot Study
Source: Front Vet Sci. 2017 Jun 8;4:83. doi: 10.3389/fvets.2017.00083 (PMC5463535; doi:10.3389/fvets.2017.00083)

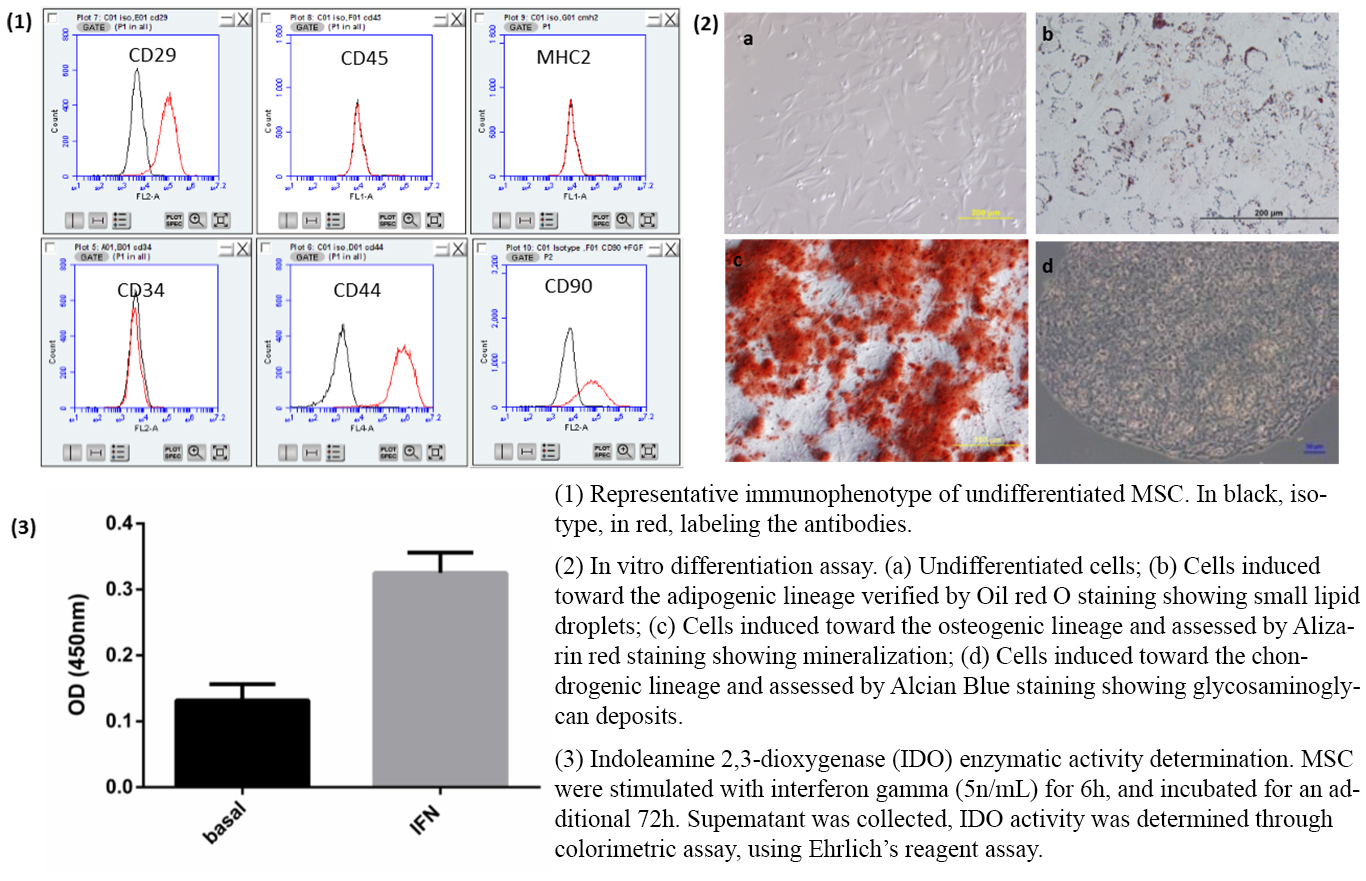

Supplement: Supplementary file 1 [file image_1.jpeg]
